# Enterovirus A71 Promotes Exosome Secretion by the Nonstructural Protein 3A Interacting with Rab27a

**DOI:** 10.1128/spectrum.03446-22

**Published:** 2023-02-15

**Authors:** Jing Wu, Yuxue Zhao, Qiaoqiao Chen, Yiwen Chen, Jiaqi Gu, Lingxiang Mao

**Affiliations:** a Department of Laboratory Medicine, Affiliated Kunshan Hospital of Jiangsu University, Kunshan, Jiangsu, China; Quest Diagnostics

**Keywords:** 3A, Rab27a, enterovirus, exosome secretion, nonstructural protein

## Abstract

Exosomes are small membrane-bound vesicles which are intraluminal vesicles (ILVs) secreted to the extracellular space after multivesicular bodies (MVBs) fuse with the plasma membrane. Although it is known that exosomes play a multitude of roles during viral infection, the mechanism that regulates their secretion during viral infection is unknown. Here, we found that enterovirus A71 (EV-A71) infection increased exosome secretion both *in vivo* and *in vitro*. Importantly, the expression of nonstructural protein 3A was sufficient to promote exosome secretion, while a mutation affecting the amino acid 18 position abrogated this effect, without changing the size of exosomes *in vivo* or *in vitro*. Transmission electron microscopy (TEM) analysis revealed that 3A decreases the number of MVBs and ILVs *in vivo* and *in vitro*, which suggested 3A may boost the fusion between MVBs and the plasma membrane. Furthermore, we demonstrated that an interaction between 3A and the small GTPase protein, Rab27a, protected Rab27a from ubiquitination, resulted in increasing exosome release. Data indicated a novel mechanism by which EV-A71 3A modifies exosome secretion during viral infection.

**IMPORTANCE** Research has shown that viral infection impacts exosome secretion, but its regulation mechanisms remain poorly understood. Nonstructural protein 3A of EV-A71 interacts with many host factors and is involved in the remodeling of cellular membranes. In this investigation, we applied exogenous expression of 3A protein for exploring its regulation on exosome secretion and utilized immunoprecipitation combined with proteomics approaches to identify 3A-interacting factors. Our results demonstrate that 3A protein upregulates the release of the exosomes and that the 3A mutant strain of EV-A71 induce less exosome release compared with the EV-A71 wild type. Viral 3A protein interacts with the host factor Rab27a to prevent it from being ubiquitinated, which in turn improves exosome secretion both *in vitro* and *in vivo*. EV-A71 3A protein is a novel viral factor in the control of exosome production.

## INTRODUCTION

Enterovirus A71 (EV-A71), a member of the *Enterovirus* genus in the *Picornaviridae* family, represents a persistent global public health threat, causing hand, foot, and mouth disease (HFMD) among infants and young children (1). HFMD may lead to severe neurological complications, such as encephalitis, aseptic meningitis, acute flaccid paralysis, and even death. EV-A71 is a nonenveloped virus with a single-stranded positive-sense genomic RNA genome of approximately 7.4-kb nucleotides. This encodes a single polyprotein that is proteolytically cleaved into four structural proteins (VP1, VP2, VP3, and VP4) and seven nonstructural proteins (2A, 2B, 2C, 3A, 3B, 3C, and 3D) (2). The structural proteins form the viral capsid and participate in viral entry, while the nonstructural proteins play a role in viral RNA transcription, translation, and replication (3). Nonstructural protein 3A is a hydrophobic protein located at the RNA replication complex and is required for viral RNA replication (4).

Exosomes, small vesicles with a diameter of 30 to 150 nm, can deliver bioactive substances, proteins, lipids, and nucleic acids, to distant cells or tissues. They are known to participate in both physiological and pathological processes (5–8). The intricate relationship between viruses and exosomes is part of a cross talk between pathogen and host. The importance of this interaction has been investigated in infections caused by the naked viruses hepatotropic viruses (HAV) and hepatitis E virus (HEV) (9, 10). In addition, there is accumulating evidence that viruses can hijack and manipulate exosomal pathways for their own benefit. Observations of this behavior were made in human herpesvirus 6 (HHV-6) (11), herpes simplex virus 1 (HSV-1) (12), Epstein-Barr virus (EBV) (13), cytomegalovirus (14), and HEV (15) infections. Our previous work showed that EV-A71-infected rhabdomyosarcoma (RD) cells secrete viral RNA packaged in exosomes, resulting in a productive infection. This mechanism of viral spread can render immune defenses less effective, facilitating the dissemination of the virus (16).

Exosomes originate as intraluminal vesicles (ILVs) contained in multivesicular bodies (MVBs). They are released to the extracellular environment upon the fusion of MVBs with the plasma membrane (PM) (17–19). Several Rab GTPase proteins, including Rab27, Rab11, and Rab25 control the necessary intracellular vesicle transport, docking, and budding (20–22), while Rab27a and Rab27b have been shown to allow docking of MVBs to the PM (23).

Recent studies demonstrated that EV-A71 infection increased exosome secretion by the monocytic leukemia cell line (THP-1) and human colorectal cell line (HT-29) (24). However, the molecular mechanism regulating exosome release remains unclear. Nonstructural protein 3A is a conserved molecule of the *Enterovirus* genus, playing a key role in the formation of viral replication complexes. The 87-amino-acid-long protein contains a C-terminal hydrophobic anchor and a soluble N terminus. The C terminus consists of a membrane domain responsible for membrane insertion, while the N terminus mainly affects the cellular protein secretory pathway via its interactions (25–28). Based on the above, the expression of exogenous 3A protein and the 3A mutant of EV-A71 virus was applied for exploring if 3A protein could impact the secretion of exosomes during the period of viral infection. In this study, exogenously introduced 3A alone was sufficient to increase exosome secretion, while the number of MVBs and ILVs within MVBs decreased. Mutations affecting 3A dramatically decreased the effect of the molecule on the secretion of exosomes both *in vitro* and *in vivo*. Moreover, we found that 3A interacted with Rab27a directly, preventing the latter from becoming ubiquitinated. This interaction appeared necessary for increasing exosome secretion. In summary, we found that during EV-A71 infection, the 3A protein induces exosome secretion by stabilizing the small Rab27a GTPase, protecting it from degradation.

## RESULTS

### EV-A71 infection promoted exosome secretion *in vitro* and *in vivo*.

**FIG 1:**
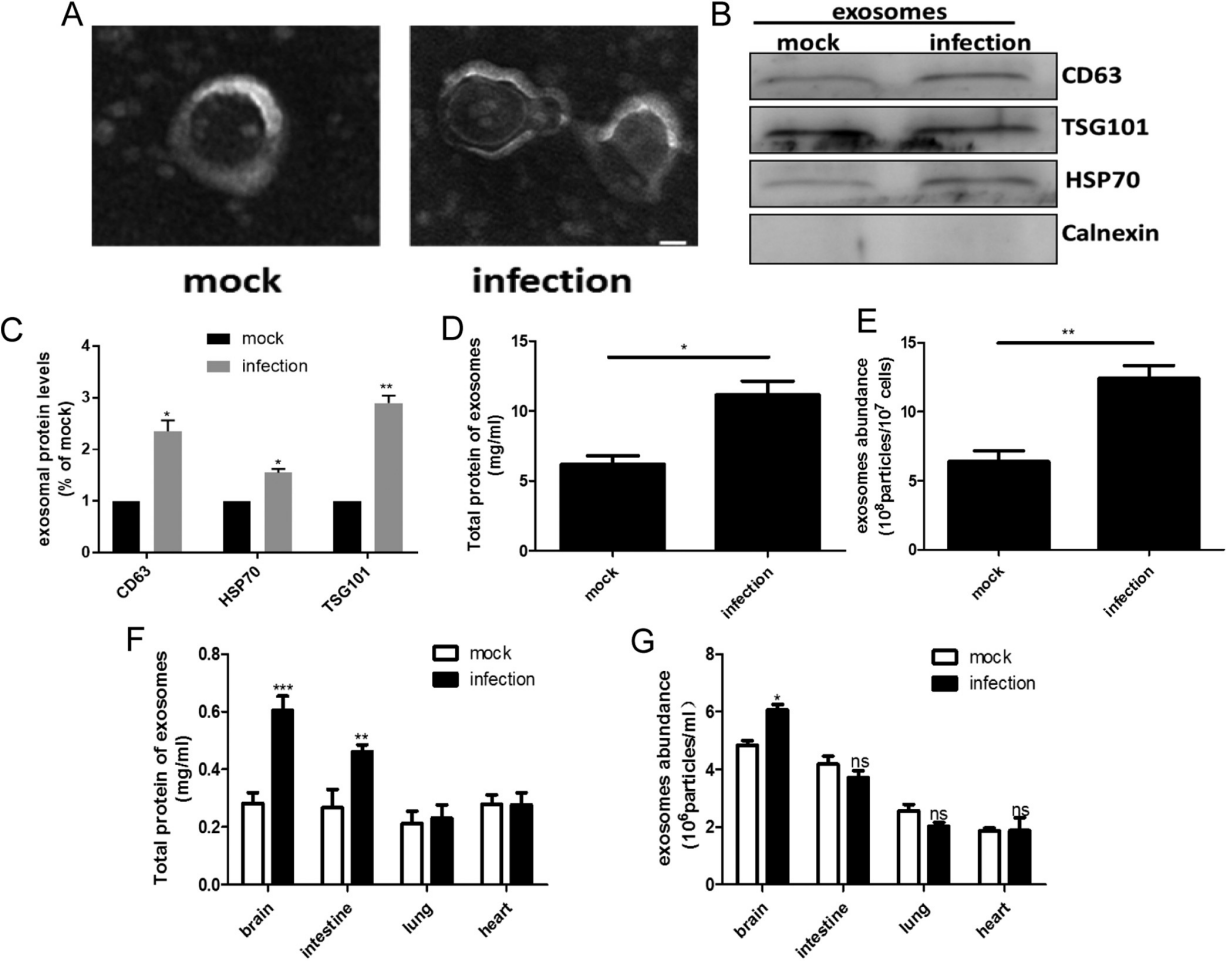
EV-A71 infection increased exosome secretion *in vitro* and *in vivo*. Exosomes were isolated and purified by serial centrifugation from supernatants of EV-A71- and or mocked-infected HT-29 cells (MOI, 1; postinfection time, 24 h). (A) Representative TEM images of exosomes. Scale bar = 100 nm. (B) Exosomal and cellular marker proteins detected by WB analysis. (C) Quantification of exosomal protein levels using ImageJ software. (D) The total exosomal proteins as detected by BCA assay. (E) Quantitation of exosomes using the Exo ELISA kit sensitive for CD63. (F and G) Neonatal ICR mice were injected with 10^6^ TCID_50_/mL of virus by intraperitoneal injection (*n* = 20) and sacrificed on day 5. Total exosomal proteins and abundance from brain, intestine, lung, and heart tissues were analyzed by BCA assay (F) or CD63 ELISA (G). Figures represent three independent experiments containing 3 samples in each group. *, *P* < 0.05; **, *P* < 0.01; ***, *P* < 0.001; ns, not significant.

The human colorectal cell line HT-29 was chosen as a cellular model for this study, as it is an intestinal cell line that can readily be infected with EV-A71. Viruses with a multiplicity of infection (MOI) of 1 were used to infect cells for 24 h, and exosomes were isolated from the cellular supernatants of EV-A71- and mock-infected HT-29 cells. TEM imaging of the collected samples showed the typical lipid bilayer morphology generally ascribed to exosomes (Fig. 1A). Nanoparticle tracking analysis (NTA) indicated that the exosomes derived from EV-A71-infected cells were similar in size to those obtained from mock-infected cells (see Fig. S1A and B in the supplemental material). Western blotting (WB) experiments detected the presence of characteristic exosomal marker proteins, including the tetraspanin cluster of differentiation 63 (CD63), heat shock protein 70 (HSP70), and tumor suppressor gene 101 protein (TSG 101), while cellular markers, for example calnexin, an endoplasmic reticulum protein, were absent. Cellular GAPDH levels were measured to standardize cell numbers at the point of harvesting the supernatants. A significant increase of the exosomal markers CD63, TSG101, and HSP70 was seen in exosomes derived from EV-A71-infected HT-29 cells (Fig. 1B and C). To investigate further whether EV-A71 infection increased the secretion of exosomes, the total protein content of the purified exosome fraction was measured and the number of exosomes was quantitated by a CD63 enzyme-linked immunosorbent assay (ELISA). Both of these approaches detected a marked increase in the number of exosomes in the cultures of EV-A71-infected HT-29 cells (Fig. 1D and E). To verify the role of viral infection in promoting exosome secretion, the same experiments were repeated using HeLa cells. As shown in Fig. S1C to F, the abundance of total proteins and the number of exosomes detected by ELISA increased significantly in EV-A71-infected HeLa cells, confirming the findings seen in HT-29 cells. To exclude the influence of cytopathic effect (CPE) on exosome release, viruses were used at a low MOI of 0.1 to preserve cellular integrity and viability. Furthermore, exosomes containing supernatants were collected from the cultures of EV-A71- and mock- infected HT-29 cells after 18 h as suggested previously (29). A similar cellular activity shown in Fig. S1G excluded the influence of cell lysis or cell death on exosome release. Increases of total proteins, protein markers, and concentration were observed in the exosome fraction of EV-A71-infected cells, further supporting the influence of this infection on exosome secretion (Fig. S1H to J). These results indicated that EV-A71 infection promoted exosome secretion in both HT-29 and HeLa cells without affecting the size of the released exosomes.

To examine whether EV-A71 enhanced the secretion of exosomes during *in vivo* infection, a mouse model was established. The virus was administered via intraperitoneal injection, and the release of exosomes was assessed in different tissues 5 days after the initial exposure. Significantly increased exosomal total protein content and exosome abundance were observed in the brain but not in the heart and lungs of the infected mice (Fig. 1F and G). Interestingly, more viruses accumulated in brain tissue than in the lung and heart (Fig. S2A and B). The CPE was also more apparent in the brains of infected animals (Fig. S2C). Taken together, these results suggested that EV-A71 infection promoted exosome secretion both *in vitro* and *in vivo*.

### Exogenous 3A enhanced exosome secretion.

**FIG 2:**
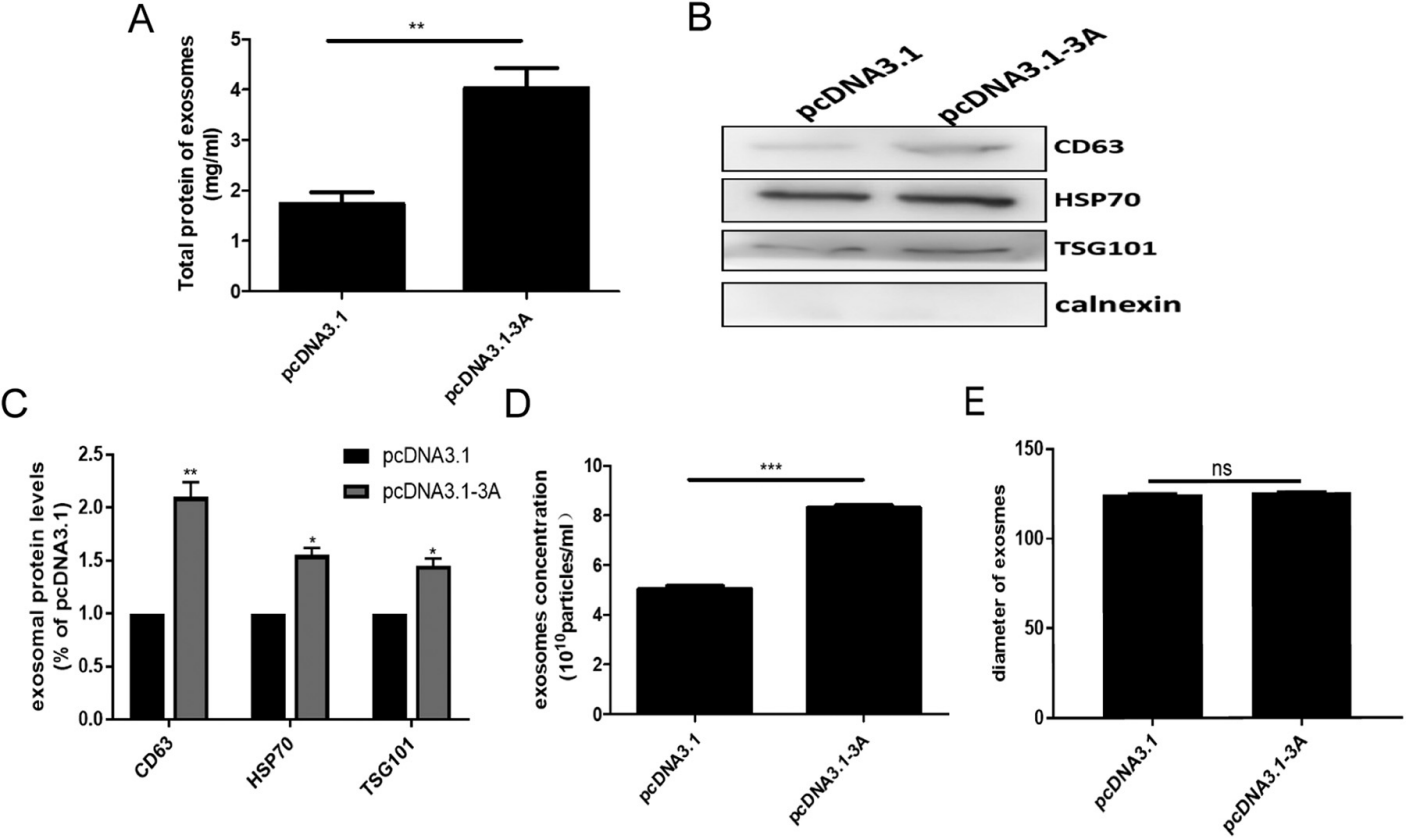
Exogenous 3A-enhanced exosome secretion. HeLa cells were transfected by a pcDNA3.1 construct carrying the full-length 3A cDNA (pcDNA3.1-3A) or the empty pcDNA3.1 vector (pcDNA3.1), and exosomes were isolated from the supernatants. (A) Total protein content of the exosomes detected by BCA assay. (B) Detection of exosomal markers using WB analysis. (C) The exosomal protein levels were quantified using the ImageJ software. (D and E) NTA analysis was carried out to compare the abundance (D) and the size (E) of exosomes. Results were derived from three independent experiments with 3 samples in each group. *, *P* < 0.05; **, *P* < 0.01; ***, *P* < 0.001.

The nonstructural protein 3A of EV-A71 is an important membrane-associated molecule (30). To investigate the possible effect of 3A on exosome secretion plasmid vectors, including the empty pcDNA3.1 vector, and a construct coding the 3A protein, pcDNA3.1-3A, were transfected into HeLa cells. After selection in G418-containing medium, the high stable expression of 3A in HeLa cells line was verified by immunofluorescence analysis (IF), WB, and flow cytometry (Fig. S3A to C). In addition, the lack of toxicity of the exogenous 3A protein was demonstrated (Fig. S3A). Exosome secretion by the transfected cells was confirmed by bicinchoninic acid (BCA), WB, and NTA analysis. The number of total exosomal proteins was found to be markedly increased in cells expressing the 3A protein (Fig. 2A). The production of exosomal markers (CD63, HSP70, TSG101) also increased following transfection by the pcDNA3.1-3A vector (Fig. 2B and C). NTA analysis also revealed an increased number of exosome particles being produced by the transfected cell line (Fig. 2D). In accordance with the observations in infected cells, there was no discernible difference in the size of exosomal particles between the 3A-transfected cells and those transfected with the empty vector (Fig. 2E). These data clearly illustrated that the introduction of exogenous 3A protein was sufficient to enhance exosome secretion in HeLa cells.

### The introduction of mutation impaired the ability of the EV-A71 3A protein to promote exosome secretion.

**FIG 3:**
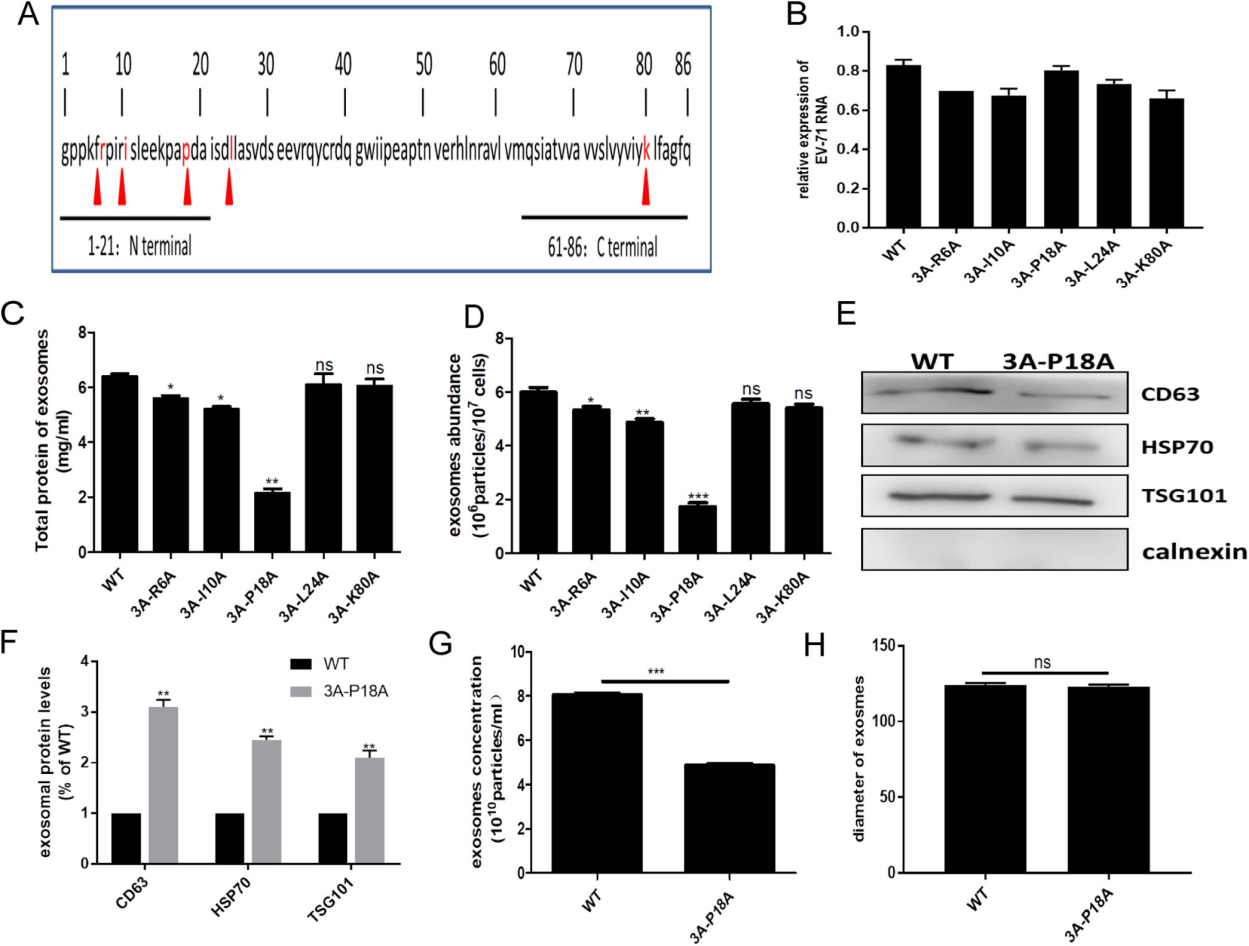
Effect of EV-A71 3A mutations on exosomes production. (A) Schematic diagram of the 3A protein highlighting the position of amino acids where mutations were introduced. (B) The level of RNA expression of the WT or mutated viruses in infected HeLa cells was determined by qRT-PCR analysis. (C) The total protein content of exosomes was detected by BCA assay. (D) The abundance of exosomes derived from WT or mutated virus-infected cells was detected using the ExoELISA kit. (E) WB analysis of exosomal markers in the supernatants of cells infected by the WT virus or the 3A-P18A mutant strain. (F) Quantitation of exosomal proteins with ImageJ software analysis of the image in panel E. (G and H) The exosome abundance (G) and exosomal diameter (H) were determined by NTA analysis. All results are from three independent experiments containing 3 replicates in each group. *, *P* < 0.05; **, *P* < 0.01; ***, *P* < 0.001; ns, not significant.

The N-terminal segment of the 3A protein, including amino acids between positions 1 and 21, is essential for interactions with cellular proteins, while those close to the C terminus, at positions 80 to 86, are necessary for membrane insertion and protein folding. Given the known relevance of these amino acids, a panel of five mutants of the full-length EV-A71cDNA clone was constructed. The positions of the mutations and the introduced amino acid changes were R6A, I10A, P18A, L24A, and K80A (31) (Fig. 3A). As the mutations were introduced into the full-length EV-A71 cDNA, after transfection into HeLa cells, the transfectants were producing viable WT or mutant viruses. The infectious particles were recovered from cultured cells and were used in subsequent experiments to infect fresh HeLa cell cultures. As described earlier, cells were infected at an MOI of 1, and supernatants were collected after 24 h. To eliminate the possibility of the mutations affecting the rate of viral replication, the amount of viral RNA was quantified by reverse transcription-quantitative PCR (qRT-PCR) (Fig. 3B). Exosomes produced by cells infected by the wild type (WT) or mutant viral strains were isolated by differential centrifugation and subsequent immune affinity purification. Viruses carrying the R6A, I10A, and P18A single-amino acid substitutions within the 3A protein did not induce exosome secretion to the same extent as WT viral particles. The P18A substitution had a particularly pronounced effect (Fig. 3C and D). Since the mutations affecting exosome secretion were all located at the N terminus of 3A, we concluded that this segment of the 3A protein was necessary for regulating the process of exosome secretion. Given the significant effect of the P18A mutation, its effect on exosome secretion was analyzed further. As shown in Fig. 3E, the expression of exosomal markers, CD63, HSP70, and TSG101, was much lower after infection with this mutant virus (Fig. 3E and F). When the supernatants were analyzed by NTA, a reduction of the number of secreted exosomes was evident in cells infected with the mutant strain of EV-A71 (Fig. 3G). However, the size of exosomes produced by cells infected by the WT or the 3A-P18A mutant virus remained unchanged (Fig. 3H).

### Exogenous 3A decreased the number of intracellular MVBs and the number of ILVs contained in them.

**FIG 4:**
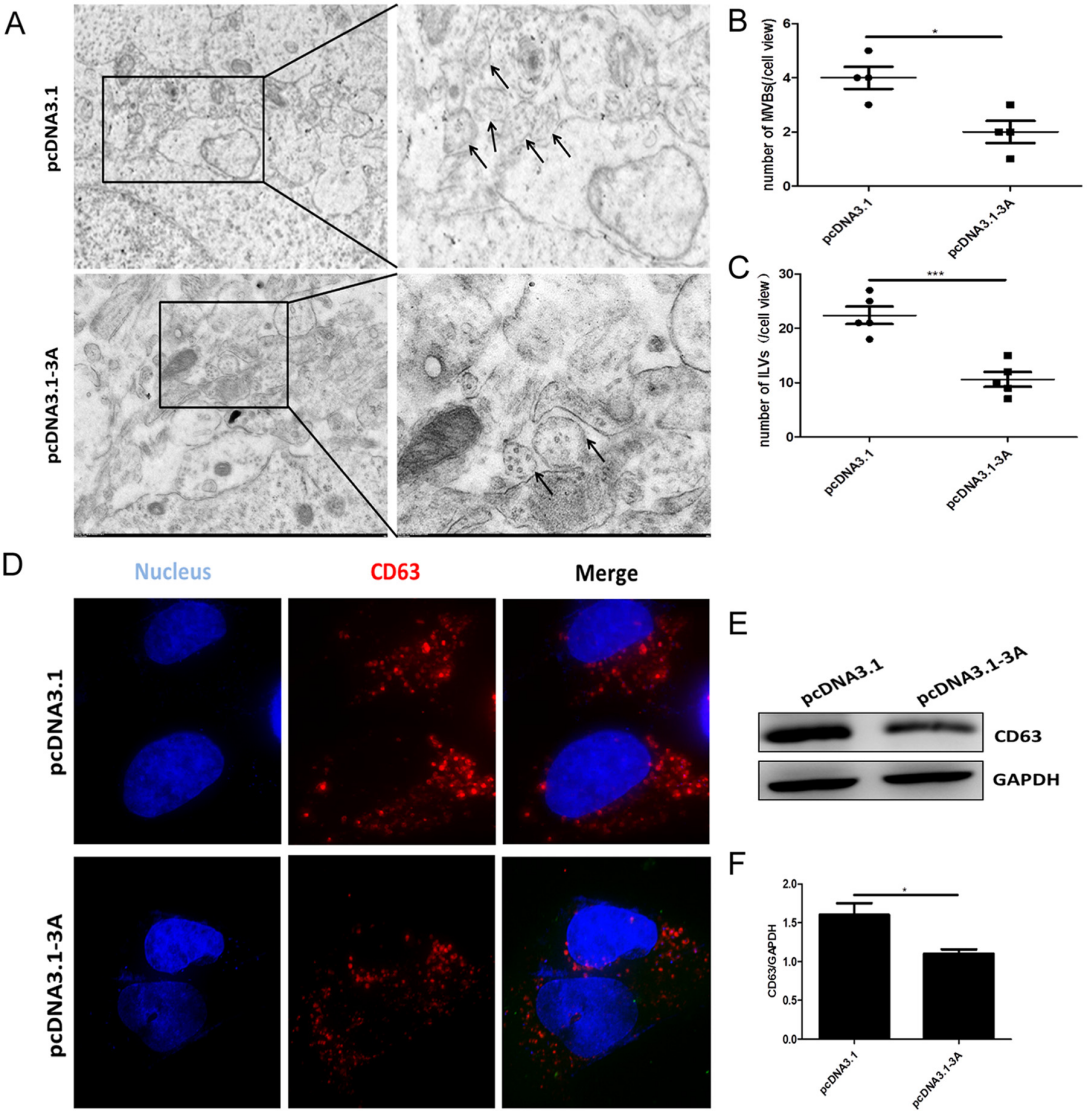
Exogenous 3A decreased the number of MVBs and the number of ILVs inside them. (A) Representative electron microscopic (EM) images of pcDNA3.1-3A and control pcDNA3.1 transfectants, showing MVBs and ILVs inside them. Scale bar = 1 μm. Enlarged sections (panels on the right) are magnified 2.6-fold. (B and C) The numbers of MVBs (B) and ILVs (C) were counted in 5 randomly selected images. (D) Representative confocal microscopic IF images of two cells stained with CD63 antibody. Red corresponds to CD63; blue corresponds to 4′,6-diamidino-2-phenylindole (DAPI) staining of the nucleus. Scale bar = 10 μm. (E) WB analysis of intracellular CD63 abundance in the pcDNA3.1- and pcDNA3.1-3A-transfected cells. (F) Graphic representation of CD63 abundance after ImageJ analysis. Expression was normalized using the abundance of the endogenous protein, GAPDH. The data shown represent three independent experiments with 3 samples in each group. *, *P* < 0.05; ***, *P* < 0.001.

To further explore the mechanism leading to increased exosome secretion in 3A-expressing cells, we investigated the number and morphology of ILVs and MVBs in HeLa cells transfected by pcDNA3.1-3A or the empty pcDNA3.1 vector. Analysis of TEM images indicated that the number of MVBs in each cell, as well as the number of ILVs in each MVB, decreased dramatically in pcDNA3.1-3A transfectants. Thus, it appeared that the presence of exogenous 3A protein led to the reduction of MVBs and the ILVs contained within them. These changes were somewhat incongruous with the increased exosome secretion seen in 3A-overexpressing cells (Fig. 4A to C). Since the size of MVBs showed no significant difference between 3A-expressing and control cells, it was possible that 3A only influenced the release of exosomes, rather than their generation.

The formation of IVLs is the result of the inward budding of endosomal membranes, resulting in formation of MVBs. The reduction of ILVs and MVBs caused by expression of the 3A protein could be due to a decreased production of ILVs or, alternatively, could be the result of their accelerated release. To distinguish between these two possibilities, we first compared the expression of Rab5 and early endosome antigen 1(EEA1), two early endosomal markers, in pcDNA3.1-3A transfectants expressing the 3A molecule and in control cells. As shown in Fig. S4A to D, IF analysis did not detect any alteration in Rab5 or EEA1 expression, suggesting that the expression of 3A did not affect the formation of endosomes and MVBs. MVBs can proceed along two distinct paths. They can either fuse with lysosomes, resulting in their degradation, or merge with the PM, leading to the release of ILVs into the extracellular space. To investigate the first possibility, we first looked at the expression of LC3B, a marker of autophagosomes. However, no change could be detected in the presence of this marker, irrespective whether 3A was expressed or not, excluding the possibility that reduced MVB numbers were due to their destruction via autophagy (Fig. S4E and F). Next, we looked at the distribution of CD63, an ILV marker, to investigate the subcellular distribution of ILVs and MVBs. IF analysis showed that the overexpression of 3A resulted in a less clustered localization of CD63 molecules (Fig. 4D). Furthermore, the expression of CD63 in pcDNA3.1-3A transfectants was lower than that in control cells (Fig. 4E and F). These results suggested that the presence of the 3A protein reduced the accumulation of ILVs inside MVBs. Based on these findings, the 3A viral protein did not affect the biogenesis or autophagy of MVBs but primarily controlled the later stages of the MVB pathway.

### The interaction of 3A with Rab27a prevents Rab27a ubiquitination.

**FIG 5:**
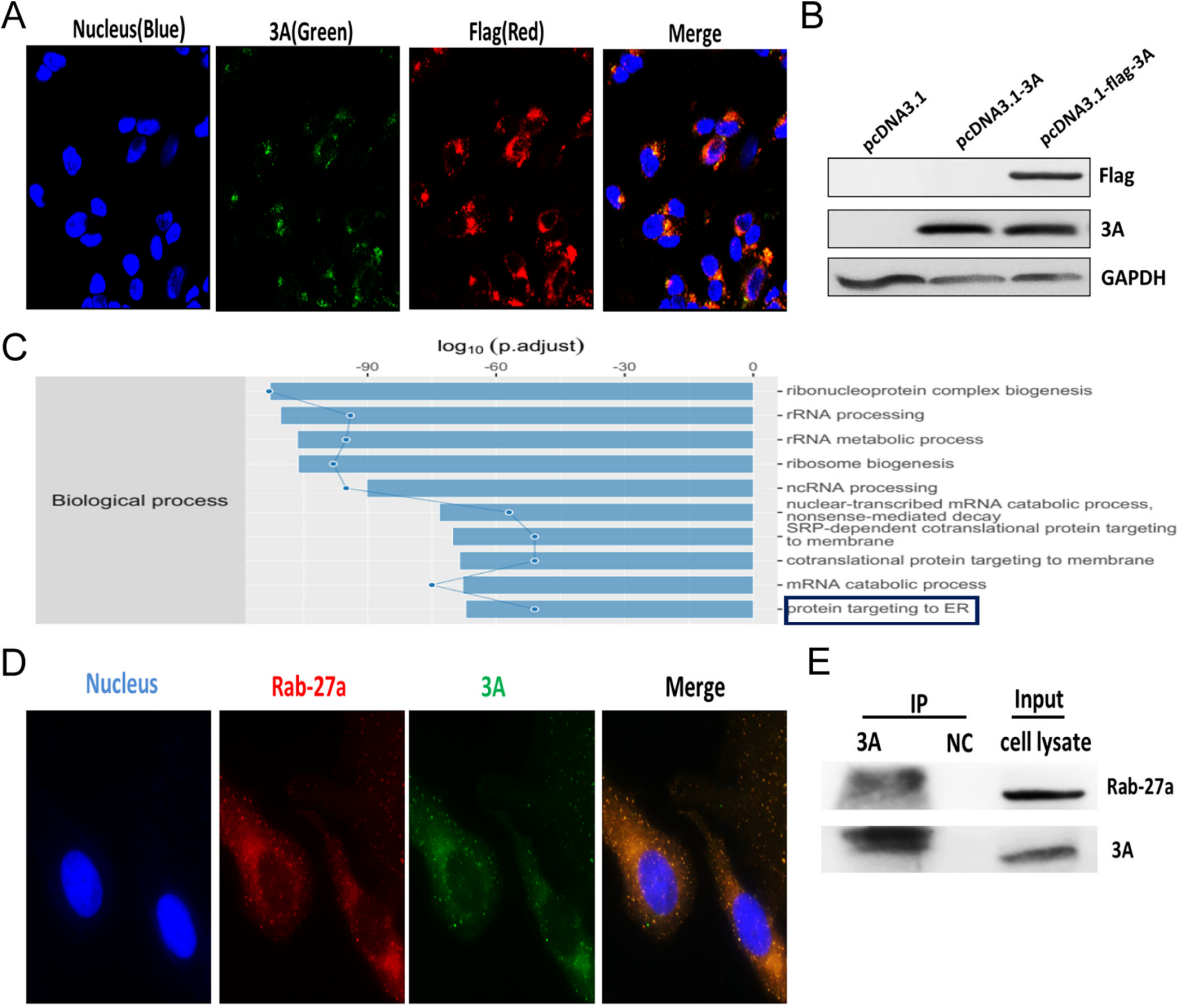
3A interacts with Rab27a directly. (A) HeLa cells were stably transfected with the pcDNA3.1-flag-3A vector, encoding a flag-tagged 3A protein. Cells were fixed and stained with anti-3A (green) and anti-flag (red) antibodies. Confocal laser scanning microscopy was performed to detect the fluorescence signal. Bar = 20 μm. (B) Samples were subjected to WB analysis using antibodies against 3A, flag peptide, and GAPDH. (C) Biological process proteins interacting with 3A were involved according to their GO classification. (D) Cells stably transfected with the pcDNA3.1-flag-3A vector were fixed and stained with anti-Rab27a (red) and anti-flag (green) antibodies. Confocal laser scanning microscopy was performed to image fluorescence signal. (E) Cells lysate was immunoprecipitated with an anti-3A antibody and immunoblotted with an anti-Rab27a antibody. These results were from 3 independent experiments with 3 samples in each group.

A flag tag epitope, consisting of the DYKDDDDK sequence, is widely used to detect recombinant proteins of interest. HeLa cells were transfected with the pcDNA3.1-flag-3A vector and analyzed using IF and WB analysis (Fig. 5A and B). To determine whether other proteins interacting with 3A are involved in exosome release, we identified proteins binding to 3A using immunoprecipitation-mass spectrometry (IP-MS) in pcDNA3.1-flag-3A cells. It was reported that 3A localized to the membranes of the endoplasmic reticulum (ER) and caused a dramatic dilation of the ER tubular morphology (32). Figure 5C shows that some of the predicted proteins interacting with 3A also localized to ER based on Gene Ontology (GO) analysis (Fig. 5C). Of the protein candidates, Rab27a was selected for further study due to its localization to the ER and known role in membrane traffic, including the fusion and release of exosomes (33). To verify the relationship between 3A protein and Rab27a, the localization of the flag-tagged 3A molecules and the endogenous Rab27a was investigated with confocal laser scanning microscopy. The results showed that Rab27a colocalized with the 3A protein in the cytoplasm (Fig. 5D). Next, we carried out an IP experiment to verify the direct interaction between Rab27a and 3A using the pcDNA3.1-flag-3A transfected cells. Rab27a was immunoprecipitated using an anti-3A antibody, while neither protein was detected in the control IgG-immunoprecipitated complex (Fig. 5E). Confocal laser scanning microscopy and IP results indicated that 3A directly interacted with Rab27a.

**FIG 6:**
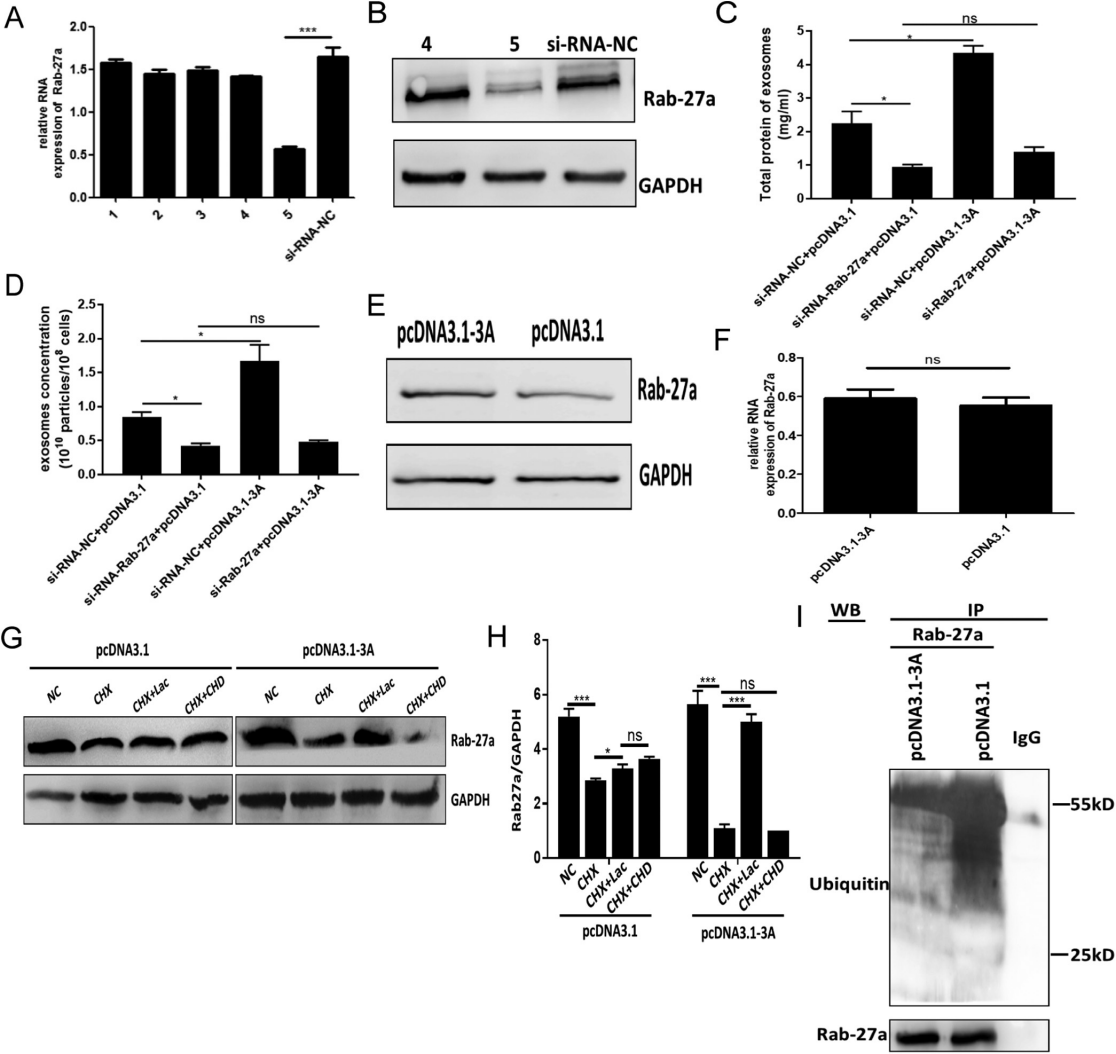
The interaction of 3A with Rab27a prevented the ubiquitination of Rab27a. (A and B) qRT-PCR (A) and WB (B) analysis of Rab27a expression in pcDNA3.1-3A cells after transfection with si-Rab27a or si-RNA-NC. (C and D) Total protein concentration in exosomes (C) and the abundance of exosomes (D) in pcDNA3.1 and pcDNA3.1-3A cells transfected with si-Rab27a or si-RNA-NC. (E) Rab27a protein levels in cell lysates using WB analysis. Normalization of Rab27a to endogenous GAPDH protein levels was carried out using ImageJ software. (F) Rab27a mRNA levels were measured by qRT-PCR analysis. The endogenous gene GAPDH was utilized as a housekeeping gene to normalize Rab27a expression. (G) WB analysis of Rab27a levels in cells treated with CHX for 12 h. In some experiments the medium was also supplemented with the proteasome inhibitor Lac or the lysosome inhibitor CHD. NC, nontreated control cells. (H) Normalization of Rab27a with endogenous protein GAPDH was carried out with ImageJ software. (I) pcDNA3.1 and pcDNA3.1-3A cells treated with Lac for 12 h before IP using anti-Rab27a or a control IgG antibody. The ubiquitinated Rab27a level was detected by WB using an anti-ubiquitin antibody. All immunoblot data represent 3 independent experiments. *, *P* < 0.05; ***, *P* < 0.001; ns, not significant.

To determine whether 3A promoted exosome secretion by interacting with Rab27a, a series of Rab27a-si-RNA (No. 1-5) constructs and a small interfering RNA (si-RNA)-negative control (si-RNA-NC) were transfected into pcDNA-3A cells. The most effective si-RNA (No. 5) was chosen for further studies (Fig. 6A and B). We found that the knockdown of Rab27a protein expression disrupted the regulation of exosome secretion by 3A protein (Fig. 6C and D).

To explain the mechanism of how the interaction between 3A and Rab27a influenced exosome secretion, we first compared the abundance of Rab27a in pcDNA3.1-3A and pcDNA3.1 cells. The result indicated that Rab27a protein expression was elevated in the presence of exogenous 3A (Fig. 6E). However, qRT-PCR analysis detected unchanged Rab27a mRNA levels (Fig. 6F), indicating that 3A controlled Rab27a protein abundance posttranscriptionally. To explore the potential mechanism by which 3A influenced Rab27a protein levels, pcDNA3.1-3A and pcDNA3.1 cells were treated with cycloheximide (CHX), a protein synthesis inhibitor, Lac, a proteasome inhibitor, and chloroquine diphosphate (CHD), a lysosome inhibitor. As shown in Fig. 6G and H, a pronounced degradation of Rab27a was detected when cells were treated with CHX for 12 h. However, the degradation of Rab27a could be reversed by the proteasome inhibitor Lac, but not by the lysosome inhibitor CHD. Taken together, these results indicated that 3A modulated Rab27a protein abundance through a protease pathway. Subsequent IP investigations revealed that ubiquitination levels of Rab27a decreased in 3A-expressing cells (Fig. 6I), indicating that 3A stabilized Rab27a by preventing its ubiquitination. Taken together, these findings indicated that the EV-A71 3A protein protected Rab27a from being ubiquitinated, extending its intracellular half-life. This in turn resulted in the increased fusion of MVBs with the PM, accelerating the release of exosomes.

### 3A-P18 is involved in the viral facilitation of exosome release through a Rab27a-dependent mechanism.

**FIG 7:**
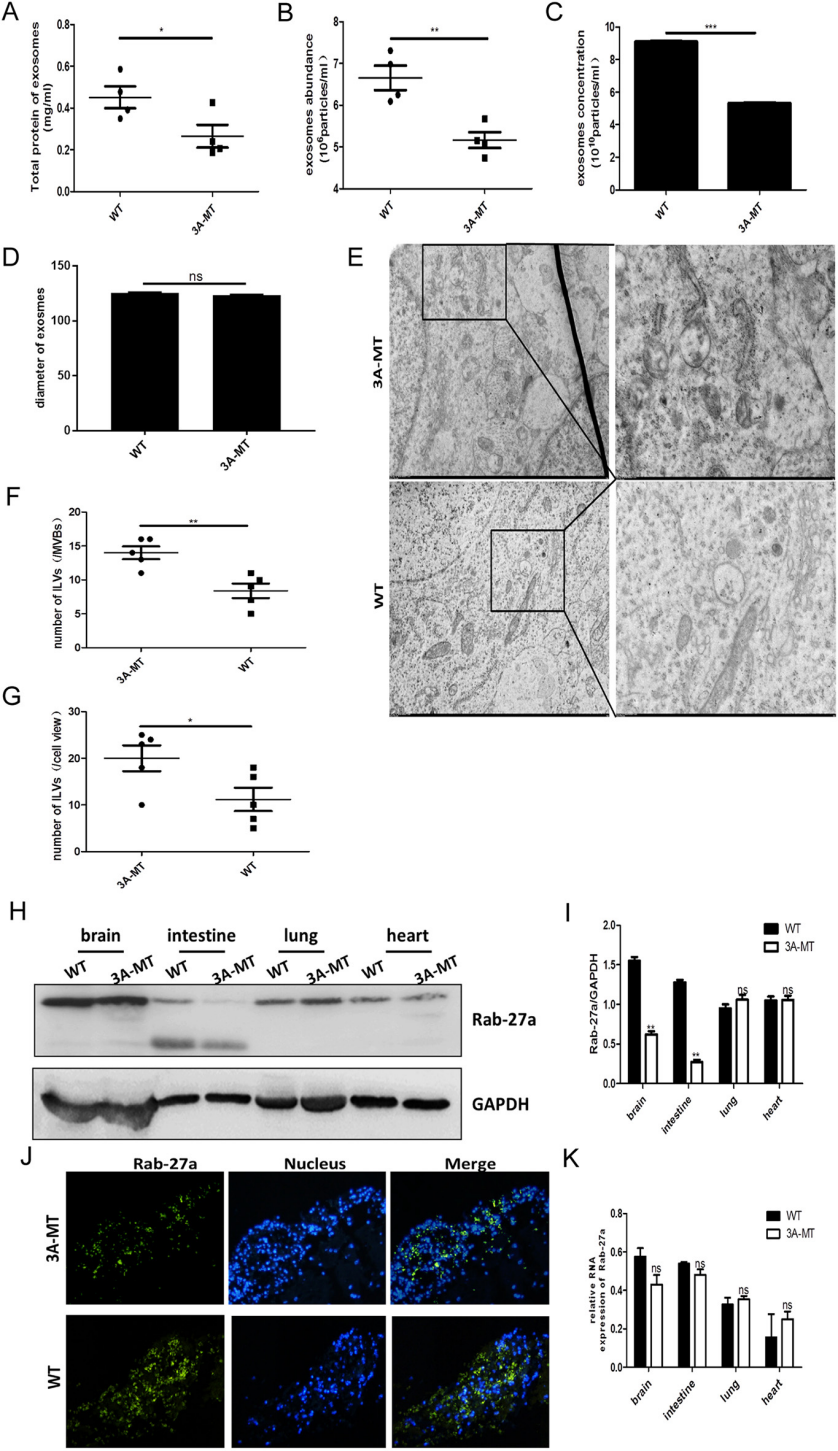
3A-P18 is important in the facilitation of exosome release via a Rab27a-dependent mechanism. (A to D) Exosomes were isolated by serial centrifugation from brain tissues of animals infected with wild-type EV-A71 virus (WT) or a mutant strain carrying an amino acid substitution at position 18 of the 3A protein (3A-MT). (A) Total protein concentration of purified exosomes detected by BCA assay. (B) The abundance of exosomes as detected by the ExoELISA kit. (C and D) NTA was performed to quantify exosomes in three independent experiments, with 10 samples in each treatment group. (C) The number of exosome particles. (D) Comparison of exosomal diameter between the two treatment groups. Particle numbers and percentage distribution are shown from representative NTA traces. (E) Representative TEM images of MVBs and ILVs. Scale bar = 1 μm. Enlarged sections (panels on the right) are enlarged 2.6-fold. (F and G) The number of MVBs and ILVs in MVBs. MVBs and ILVs in 5 typical randomly selected images were counted. (H) WB analysis of Rab27a protein expression in the brain, intestine, lung, and heart of 3A-MT mice and WT mice. (I) Rab27a protein expression normalized to the abundance of the GAPDH internal control. (J) IF staining showing Rab27a expression in the brain of 3A-MT mice and WT mice. (K) qRT-PCR analysis of Rab27a mRNA abundance in various tissues of 3A-MT and WT mice. These results represent three independent sets of experiments with 3 samples in each group. *, *P* < 0.05; **, *P* < 0.01; ***, *P* < 0.001; ns, not significant.

After observing the effect of the EV-A71 3A mutations on exosome production *in vitro*, we decided to investigate the effect of the mutations in a more clinically relevant *in vivo* animal model. Neonatal mice were infected with the EV-A71 WT and the mutant EV-A71 3A-P18A virus (for brevity referred to as WT mice and 3A-MT mice in the following section). Brain tissue was isolated from the infected animals, and exosomes were isolated by serial centrifugation steps. Consistent with the findings of *in vitro* experiments, both the total protein content and the abundance of secreted exosomes were significantly lower in the brain tissue of 3A-MT mice (Fig. 7A and B). Additional NTA analysis also confirmed that a reduced number of exosomes was produced in the brain tissue of 3A-MT animals (Fig. 7C). In concordance with previous experiments, there was no difference in the diameter of exosomes between the two groups (Fig. 7D). Next, we visualized the detailed structure of MVBs and ILVs in brain tissue of 3A-MT mice and WT mice using TEM. The obtained images showed an increase of the number of LIVs per MVB in 3A-MT mice compared to WT mice, along with the increase in the number of MVBs (Fig. 7E to G). These results suggested that a single amino acid change replacing the proline residue at P18 of the 3A protein could damage the viral stimulation of exosome secretion *in vivo*.

To examine the molecular mechanism utilized by the 3A protein to regulate exosome secretion *in vivo*, the amount of Rab27a was compared in brain, intestinal, lung, and heart tissues of 3A-MT and WT mice using WB. In these comparisons the amount of Rab27a was significantly lower in the brain and intestine of animals infected with the mutant virus (Fig. 7H and I). Since the most pronounced differences in Rab27a levels were seen in the brains of the animals, the tissue where EV-A71 replicated the most rapidly (Fig. S2), we conducted additional IF observations in brain tissue. The images showed that the brains of animals infected with the mutated 3A virus contained lower levels of Rab27a protein (Fig. 7J), while Rab27a mRNA abundance remained unchanged. These qRT-PCR experiments also showed that the mRNA level was unchanged in all other tissues, irrespective of whether the animals were infected with WT or mutated viruses (Fig. 7K). Thus, the *in vivo* results reproduced the findings of *in vitro* experiments, supporting both the critical role of the proline residue at P18 of the 3A protein in regulating exosome secretion and the stabilization of Rab27a expression seen in cells infected with the WT virus.

### Exosome secretion induced by the 3A protein impacted the infectivity of EV-A71.

**FIG 8:**
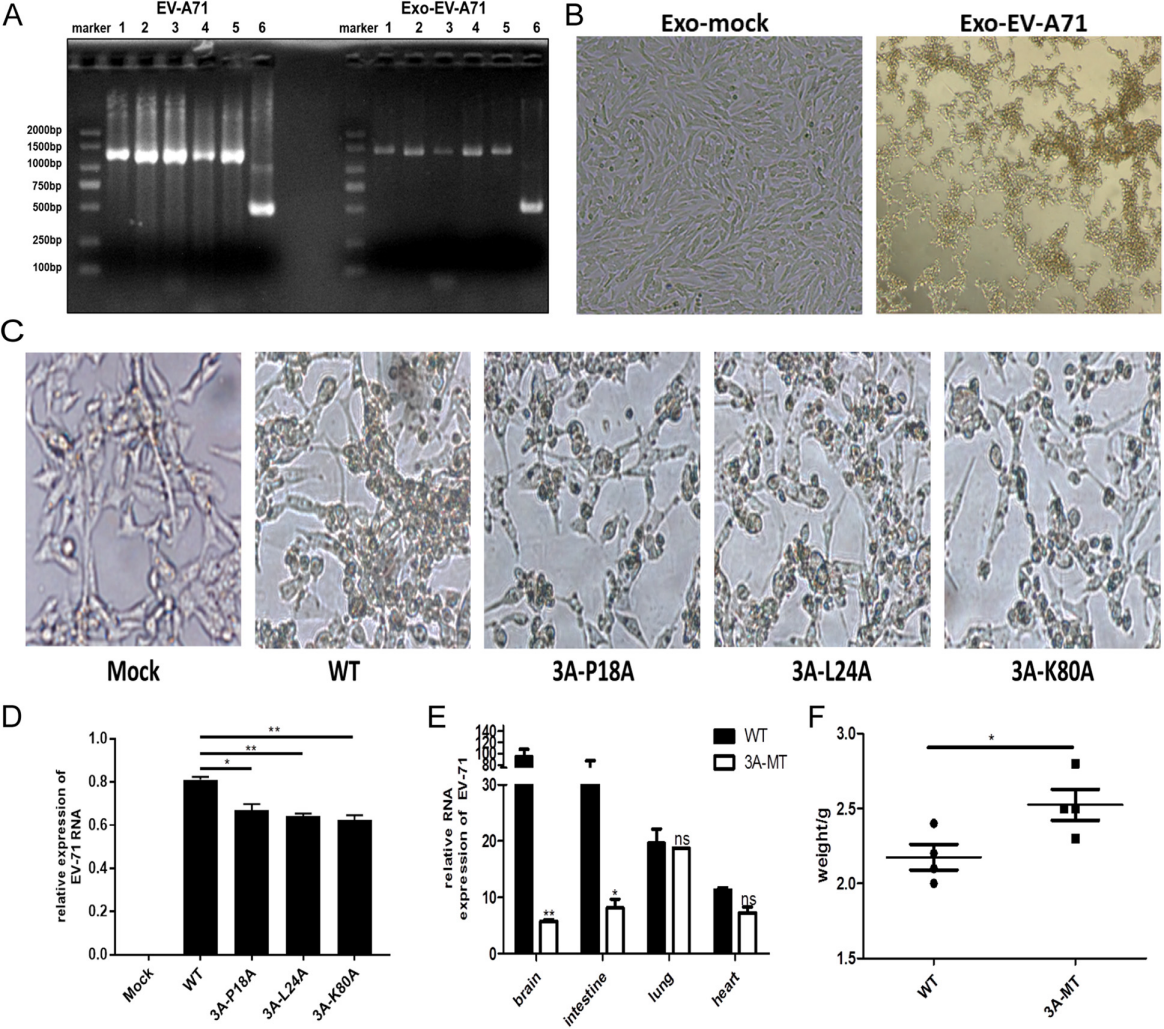
The mutation of 3A causing reduced exosome release interfered with the infectivity of EV-A71 *in vitro* and *in vivo*. (A) The segmented PCR product of viral RNA in exosomes was identified by agarose gel electrophoresis. The EV-A71 virion was used as a positive control. (B) RD cells were treated with exosomes derived from mock-infected cells (Exo-mock) or Exo-EV-A71 for 24 h. CPE in RD cells caused by the infection of Exo-EV-A71 was detected. (C and D) RD cells were treated with infectious rescued viral strains EV-A71-WT, 3A-P18A, 3A-L24A, and 3A-K80A for 24 h. (C) CPE in RD cells caused by the infection of different viral strains. (D) Viral RNA abundance level in RD cells was detected by qPCR. (E) qRT-PCR analysis of the abundance of viral RNA in the brain, intestinal, lung, and heart tissues of 3A-MT mice infected with the WT virus or the strain carrying the P18A mutated version of the 3A protein (3A-MT). (F) The weight of mice recorded on the fifth day after infection. Each group consisted of 5 animals. *, *P* < 0.05; **, *P* < 0.01; ns, not significant.

We have previously shown that exosomes secreted by EV-A71-infected cells can transmit viral infection, demonstrating an exosomal route of viral transmission (16). In this study, the experiments also showed that the exosomes derived from EV-A71-infected cells (Exo-EV-A71) contain full-length virus RNA (Fig. 8A). As expected, Exo-EV-A71 acted as an infectious agent on the cells, helping it establish a productive infection (Fig. 8B). To explore whether the 3A protein impacted viral infectivity *in vitro*, we exposed RD cells to WT or mutated 3A-P18A, 3A-L24A, and 3A-K80A strains of the EV-A71 virus and observed the resulting CPE. Compared to WT, CPE was reduced significantly in the mutant virus-infected cells (Fig. 8C). The analysis of RNA abundance using qRT-PCR revealed that viruses carrying any of the 3A mutations showed reduced infectivity in RD cells *in vitro* (Fig. 8D). When tested in the animal model, the abundance of the viral RNA was significantly lower in brain and intestine samples in animals infected with the 3A-P18A mutant (3A-MT mice) than in animals infected with the WT strain (WT mice) (Fig. 8E). Interestingly, these findings were consistent with Rab27a expression levels, which were also predominantly decreased in the brain and intestinal tissues in 3A-MT-injected mice. We also recorded the weight of mice after the infection and found that animals infected with the strain carrying the 3A mutation gained more weight than animals exposed to the WT EV-A71 (Fig. 8F). Together, these results indicated that mutations within the 3A protein alleviated the infections caused by EV-A71 both *in vitro* and *in vivo*. It appears that this reduced infectivity coincides with reduced exosome release by cells infected with the mutant viral strains.

## DISCUSSION

An EV-A71 infection can result in severe central nervous system (CNS) pathology, ranging from aseptic meningitis with or without pulmonary edema to brainstem encephalitis (34, 35). In a previous study we found that exosomes derived from EV-A71-infected cells carry viral RNA that was sufficient to establish a productive infection in human neuroblastoma cells (16). These infectious exosomes crossed the blood-brain barrier, facilitating viral pathogenesis in the CNS (36). There is accumulating evidence suggesting that exosomes are critical intercellular communication channels in the transmission of viruses. Furthermore, certain viruses exploit the exosomal pathway of the host for their assembly, budding, and release (37–39).

Previous studies have shown that EV-A71 infection increases exosome secretion by THP-1, HeLa, or HT-29 cell (24). The virus also induced exosome release from differentiated C2BBe1 cells and human intestinal organoid established from human embryonic stem cells (40). Here, we confirmed that EV-A71 infection can also promote exosome secretion both *in vitro* and *in vivo*.

In infected cells, the HIV Nef protein stimulates its own transport to the extracellular space via the release of exosomes. Similarly, the latent membrane protein 1 (LMP1) of EBV enhances exosome production by interacting with CD63, a late endosomal protein (13). However, the mechanisms of how viral components control exosome release remain unclear.

3A, a nonstructural protein of the EV-A71 virus, plays a critical role in the infectivity of the virus. 3A is a membrane-bound protein. Its region at the N terminus, composed of 22 amino acid residues, can directly interact with host proteins (28). It has been demonstrated that the presence of 3A proteins induces the inner membrane of cells to form intracellular vesicles. This process is favorable for viral replication but is not associated with exosome secretion. In our study, we confirmed the role of EV-A71 nonstructural protein 3A on exosome secretion and demonstrated the importance of the presence of the proline residue at position 18 at the N terminus in this process.

Exosomes are constitutively generated as the in-budding of endosomes leads to the development of MVBs containing ILVs. As MVBs fuse with the PM, ILVs are released as exosomes. In an alternative processing pathway MBVs can fuse with lysosomes, resulting in the degradation of their content (41). Our results showed that the introduction of exogenous 3A protein increases the number of exosomes without changing their size. However, the presence of the 3A protein did not induce any changes in the expression of early endosomal markers (EEA1 and Rab5) or the autophagosomal marker LC3B, suggesting that 3A promotes exosome secretion by affecting the late stages of the MVB processing pathway. To gain further insights into the molecular mechanisms, we looked for proteins interacting with 3A in cells expressing a flag-tagged form of 3A. IF and IP-MS studies in this experimental system confirmed the colocalization and direct interaction of 3A with Rab27a.

Various Rab proteins are involved throughout the life cycle of EV-A71. Ra5 and Rab7 are necessary for viral entry, Rab11 and Rab9 are involved in viral assembly, and Rab6 is involved in viral glycoprotein trafficking (42). At the same time, several Rab proteins, including Rab11, Rab27, and Rab35, have been found to participate in the transport of MVBs to the PM and subsequent exosome release (21, 43–46). However, there was no evidence of the virus using Rab GTPases for exosome biogenesis. In the experiments presented here, we showed that viral 3A protein upregulated the abundance of Rab27a at the protein level without a change in mRNA abundance, indicating that 3A regulates Rab27a expression posttranslationally. In further experiments, we detected that 3A increased Rab27a protein levels by reducing its degradation without affecting the synthesis of the mRNA or protein. In knockdown experiments the reduction of Rab27a mRNA abundance abrogated any increase in exosome secretion caused by 3A expression, indicating that Rab27a was a key mediator in this process. The N terminus of the 3A protein plays an important role in this process, as verified by the effect of several EV-A71 3A mutations in this region.

There are several issues and challenges in elucidating the complete mechanism of viral control of exosome biogenesis during EV-A71 infection. In addition to 3A, other nonstructural proteins, such as 2C, have potential membrane-binding activity and associate with ER protein of the host cells (47). It has been shown that coat protein complex I (COPI) is involved in the formation of picornavirus-induced vesicles via the 2C protein (48). Therefore, the role of other nonstructural proteins in the stimulation of exosome release needs to be explored further. However, there is a more challenging contradiction. While exosomes can disseminate an infection by transmitting viral nucleic acid components, at the same time, they also carry a large number of antiviral factors that potentially inhibit viral spread. The balance between these contradictory mechanisms may depend on the type and state of cells releasing the exosomes. The mechanisms by which viruses regulate exosome biogenesis is complicated and worthy of further investigation.

The findings presented here further substantiate previous observations that EV-A71 infection induces exosome secretion both *in vitro* and *in vivo*. We demonstrated that the 3A viral protein promotes exosome secretion by preventing the ubiquitination of Rab27a, thereby extending the life span of the existing protein molecules. These findings provide the first clues in piecing together how exosome secretion is influenced by EV-A71. Verifying the mechanism leading to increased exosome release after an infection in both cell culture and infected mice could expand our understanding of viral pathogenesis. Given the pivotal role of exosomes in various biological processes, our study may pave the way for further investigations into the role of exosome biogenesis in viral infections. This will provide a greater understanding of viral pathogenesis and may identify the therapeutic targets in combating viral diseases.

## MATERIALS AND METHODS

### Cell culture, viral culture, and animals.

The human cervical carcinoma cell line (HeLa), human colorectal cell line (HT-29), and human rhabdomyosarcoma cell line (RD) were grown in Dulbecco’s modified Eagle medium (DMEM) (Gibco, Thermo Fisher Scientific) supplemented with 10% fetal bovine serum (FBS) (Gibco, Thermo Fisher Scientific) and 1% antibiotic-antimycotic (100X; Thermo Fisher Scientific) in a 37°C humidified atmosphere with 5% CO_2_. The EV-A71 viral strain (GenBank accession number OP191657) was isolated from a throat swab of an infected patient and inoculated into RD cells for propagation. The enterovirus A71 RNA detection kit (S-SBIO, China) was used to identify the isolated virus. Cells were infected at a multiplicity of infection (MOI) of 1 in medium supplemented with 2% FBS and 1% antibiotic-antimycotic. During experiments investigating exosome release, the 10% FBS was replaced with exosome-depleted FBS (ED-FBS) (Gibco, Thermo Fisher Scientific). For the animal experiments, neonatal ICR mice were used following a protocol approved by the hospital’s ethics committee.

### Virus titration.

The 50% tissue culture infectious dose (TCID_50_) titers were determined as follows: 10^4^ RD cells were seeded in 96-well plates before infection. The virus samples were serially diluted in DMEM containing 2% FBS (10^1^ to 10^10^), and each diluted sample was added to 8 separate wells. The plates were incubated for 7 days, and the cytopathic effect (CPE) was observed under a microscope. To determine the viral titer, expressed as TCID_50_, a Reed-Muench endpoint calculation was performed (49).

### Isolation of exosomes from cultured cells.

Cell-conditioned medium was collected from EV-A71-infected and mock-infected cells cultured in DMEM containing 2% ED-FBS for 24 h. Cell-conditioned medium was collected from cells transfected with pcDNA3.1-3A or pcDNA3.1 and cultured in DMEM containing 10% ED-FBS for 48 h. The collected medium was spun at 2,000 × *g* at 4°C for 15 min to remove cellular debris and apoptotic bodies. To remove large enteroviruses (EVs), the supernatant was passed through a 0.22-μm-pore polyethersulfone (PES) filter (Millipore, USA). Next, the supernatant was centrifuged at 10,000 × *g* and 4°C for 30 min to remove any remaining large EVs. Finally, the supernatant (prepurified medium) was subjected to ultracentrifugation at 120,000 × *g* and 4°C for 24 h to collect exosomes. Purified exosomes were suspended in filtered phosphate-buffered saline (PBS) for further experiments. Cellular debris and microvesicles in the supernatant were removed through the series of centrifugation steps.

### Isolation of exosomes from mouse tissue samples.

The protocol for the isolation from brain, intestine, lung, and heart tissues was established previously. In brief, fresh frozen (–80°C) tissues were sliced into 1- to 2-cm-long, 2- to 3-mm-wide sections. The cut sections were dissociated while partially frozen in 75 U/mL of collagenase type 3 in Hibernate-E at 37°C for a total of 20 min. The tissue was returned to ice immediately after incubation, and protease and phosphatase inhibitors were added. The tissue was spun at 300 × *g* for 5 min at 4°C. The pellet was used as the brain homogenate plus collagenase control, while the supernatant was transferred to a fresh tube, spun at 2,000 × *g* for 10 min at 4°C then at 10,000 × *g* for 30 min at 4°C. This process was previously shown to result in minimal cell lysis (50). The EV-containing supernatant was overlaid on a triple sucrose gradient (0.6 M, 1.3 M, 2.5 M) and ultracentrifuged for 3 h at 180,000 × *g* to separate vesicles based on density. The top of the gradient was discarded, and fractions designated 1, 2, and 3 were collected, and their refractive index was measured. Each fraction was further ultracentrifuged at 100,000 × *g* to pellet vesicles. Each preparation was validated by a combination of techniques, including TEM, RNA, and protein analysis.

### Enrichment of exosomes by immunoaffinity magnetic beads.

An exosome-human CD63 isolation/detection kit was used to enrich CD63^+^ exosomes according to the manufacturer’s instructions (Invitrogen, USA). Briefly, 100 μL of preenriched exosome sample was incubated with 20 μL CD63^+^ magnetic beads overnight at 4°C. Exosomes were positively selected using a magnet, and samples were washed in isolation buffer to eliminate nonspecific binding. Bead-bound exosomes were resuspended in 300-μL PCR-grade water for further studies (51).

### Transmission electron microscopy.

Purified and enriched exosome pellets were resuspended in 100 μL of particle-free PBS and fixed in 4% paraformaldehyde and 4% glutaraldehyde in 0.1 M phosphate buffer (pH 7.2). Each exosome sample was moved to a carbon-coated copper grid and immersed in 2% phosphotungstic acid solution (pH 7.0) for 1 min. Fresh mouse brain tissues and cells were fixed with 2% glutaraldehyde and 1% osmium tetroxide, rinsed in 100 mM sodium phosphate buffer, dehydrated in ethanol, and embedded in Epon. Ultrathin sections of brain tissues or cells were collected on Formvar-coated grids and stained with 10% uranyl acetate and 1% lead citrate. All samples were examined using a transmission electron microscope (JEM-1200EX; JEOL Ltd., Tokyo, Japan) at an acceleration voltage of 80 kV.

### ELISAs.

Purified and enriched exosome pellets were suspended in 50 μL of coating buffer and were analyzed using CD63 Exo ELISA (EXOEL-CD63A-1) (System Bioscience, USA). Assays were performed according to the manufacturer’s instructions. Briefly, each exosomes sample was immobilized onto the wells of a microtiter plate at 37°C for 1 h. Plates were washed 3 times for 5 min with 100 μL wash buffer. Next, 50 μL of primary CD63 antibody and 50 μL of secondary antibody were added to blocking buffer, and plates were incubated at room temperature for 1 h with shaking. Plates were washed 3 times for 5 min with 100 μL wash buffer. Then, 50 μL of supersensitive TMB (*N*,*N*,*N′*,*N′*-tetramethyl-1,3-butanediamine) ELISA substrate was added to each well and the plates were incubated at room temperature for 15 min with shaking and analyzed with a spectrophotometric plate reader at 450 nm.

### Transfection and selection of positive clones.

Empty pcDNA3.1 vector (pcDNA3.1), pcDNA3.1-3A, and pcDNA3.1-flag-tagged 3A (pcDNA3.1-flag-3A) were purchased from GenePharma Company (Suzhou, China). The three plasmids, pcDNA 3.1, pcDNA 3.1-3A, and pcDNA3.1-flag-3A, were transfected into HeLa cells using Lipofectamine 2000 (Life Technologies, USA) according to the manufacturer’s instructions. After transfection, cells were cultured in DMEM containing 10% FBS and 400 μg/mL G418 for 48 h. Next,14 days later, the concentration of G418 was changed to 200 μg/mL to maintain selection pressure. G418-resistant clones became visible approximately 10 days after transfection. G418-resistant clones were picked and cultured further. Then, 4 weeks after transfection, three cell lines were obtained. Here, these will be referred to as pcDNA3.1, pcDNA3.1-3A, and pcDNA3.1-flag-3A. These cell lines were cultured in DMEM containing 200 μg/mL G418 to maintain the stable expression of the transfected vector (52).

### Construction and transfection of mutated viral strains.

The pBR322 EV-A71 (pEV-A71) vector, containing the full-length EV-A71 genome, was kindly provided by Qi Jin. Based on this, different mutations of the 3A-coding sequence were constructed by site-directed mutagenesis using PrimeSTAR GXL DNA polymerase (TaKaRa, China). After transformation and DNA extraction, plasmid vectors were linearized by SalI digestion. RNA transcripts were produced using the T7 *in vitro* transcription kit (Ambion, USA) according to the manufacturer’s instructions. After purification, both the *in vitro*-synthesized RNA transcripts and pEV-A71 plasmid were transfected into cells in 24-well plates using the Lipofectamine 2000 transfection reagent according to the manufacturer’s instructions. When more than 85% of the cells showed typical CPE, cells were exposed to 3 freeze-thaw cycles, and the rescued viruses were harvested.

### Flow cytometry.

For intracellular staining, the 3A, pcDNA3.1, and pcDNA3.1-3A cell suspensions were first stained with a mouse monoclonal anti-human antibody against 3A (anti-3A MAb) for 60 min at 4°C. After being washed 3 times in Tris-buffered saline with Tween 20 (TBST), cell suspensions were incubated with secondary antibodies for 45 min at room temperature. Following 3 more washes, cells were analyzed using an FACSCalibur flow cytometer (Becton, Dickinson, Mountain View, CA) and FlowJo software according to the manufacturer’s protocol.

### Establishing the infection model in experimental animals.

ICR mice (1 day old) were intraperitoneally injected with wild-type or mutated EV-A71 viruses containing 10^6^ TCID_50_ of viral particles. This administration of the virus led to an infection causing indirect damage to the nervous system, reproducing several features of a natural infection in humans. Infected mice were sacrificed on day 5, and brain, intestine, lung, and heart tissues were collected for further analysis. Animal experiments were approved by the institutional animal care and use committee.

### Nanoparticle tracking analysis (NTA).

NTA was used to quantify nanoparticle concentrations and the size of exosome populations. Isolated and enriched exosome samples were resuspended in particle-free PBS to obtain a recommended concentration and vortexed for 1 min. The samples were then analyzed using a NanoSight instrument (ZetaView version 8.04.02, Germany) to capture particles moving by as a result of electrophoresis and Brownian motion. The concentration, diameter, and percentage distribution of exosomes were recorded, and a particle image was captured. The quantity of particles measured by NTA was normalized to the number of cultured cells counted at the time of harvest to calculate the number of exosomes secreted by each cell.

### Immunofluorescence analysis (IF).

Cells cultured on a coverglass were fixed with 4% paraformaldehyde, washed with phosphate-buffered saline (PBS), permeabilized with 0.5% Triton X-100/PBS for 15 min, blocked in TBST buffer containing 5% nonfat dried milk, and stained with primary antibodies diluted 1:100 in PBS. The staining was carried out overnight at 4°C. Cells were then washed with PBS and incubated with secondary antibodies at room temperature for 1 h. Samples were counterstained with DAPI (4′,6-diamidino-2-phenylindole) dye and observed with a fluorescence microscope or TCS SP5 II laser scanning confocal microscope (Leica, Germany).

### Western blotting (WB) and immunoprecipitation (IP).

Cells were harvested and lysed using a radioimmunoprecipitation assay (RIPA) buffer (Kangwei Century, China). In brief, extracted proteins were separated on an SDS-PAGE gel and transferred to polyvinylidene fluoride (PVDF) membranes (0.22 μm; Bio-Rad, USA). The membranes were blocked in TBST buffer containing 5% nonfat dried milk and washed in Tris-buffered saline (TBS) with 0.01% Tween 20 (PBST). Proteins were detected with primary antibodies and peroxidase-conjugated secondary antibodies and visualized using an enhanced chemiluminescence (ECL) substrate. For IP analysis, an anti-3A antibody (kindly provided by Jianwei Wang) or IgG control antibody (Cell Signaling Technology, USA) was added to each sample in RIPA buffer and incubated overnight at 4°C with rotation. The next day, protein G magnetic beads (Thermo Fisher Scientific, USA) were added to the sample/antibody mixtures and incubated with rotation at room temperature, and proteins were prepared for WB analysis.

### RNA extraction and analysis.

Total RNA was extracted from cells and tissues using TRIzol reagent (Invitrogen, USA) following the manufacturer’s instructions. The RNA was reverse transcribed using the PrimeScript RT reagent kit (TaKaRa, Japan) and amplified with Luminaris Color HiGreen qPCR master mix (Thermo Fisher Scientific, USA). GAPDH mRNA levels were used as an internal control to normalize RNA expression. Primers, synthesized by Sangon Biotech (Shanghai, China), were as follows: 5′-GCCATAGCACTCGCAGAGAA-3′ (forward) and 5′-TGTCCACACACCGTTCCATT-3′ (reverse) for Rab27a. Primers of GAPDH were purchased from Sangon Biotech. The sequence of the EV-A71 primers (forward, 5′-AGGATTTACATGAGAATGAAGCA-3′; reverse, 5′-GCATAATTTGG GTTGGCTTT-3′) were previously published (16). Relative RNA expression was calculated using the comparative threshold cycle method and determined by value of 2^–ΔΔ^*^CT^*. Reverse transcription-PCR (RT-PCR) was performed using PrimeScript one-step RT-PCR kit version 2 (TaKaRa Bio, Inc., Japan) per the manufacturer’s instructions. The 7 pairs of primers used for detecting EV71 RNA in exosomes are presented in Table 1.

**TABLE 1:** RT-PCR primers used in this study

Amplified fragment of viral RNA	Primer sequence
1-1316F	TTAAAACAGCCTGTGGGTTGC
1-1316R	GGAGTGCTCCTTGATGGAATTTA
1184-2485F	TACTGGAAGTTCCCGGATGTGT
1184-2485R	CACGCTATCTCCTATGGAGCTTT
2310-3606F	CAAATTATGTGGTTCCAATCGGT
2310-3606R	CTGGCTGGGTAACACTCGCTA
3419-4731F	TCTTGTCTGGGAAGACAGCTCTC
3419-4731R	TGACAAACTTAGAGGTGAAGGAAACT
4547-5865F	CCACCAGACCCGGATCATT
4547-5865R	ATACCGATAACCTTCCCAACAGAT
5641-7095F	ATATCACCAAATTCATCCCAGAGA
5641-7095R	GAAACTGTTCATCGGGCAAAA
6903-7405F	ACATGGTTGCTTATGGAGACGAT
6903-7405R	GCTATTCTGGTTATAACAAATTTACCC

### Immunoprecipitation-mass spectrometry (IP-MS).

Harvested cells (>2 × 10^7^) transfected with pcDNA3.1-flag-3A or empty pcDNA3.1 vectors were prepared for IP according to the guidelines for collection and preservation for Aksomics biological samples. The IP-MS was carried out by Aksomics, Inc. (Shanghai, China). Briefly, after lysis in PBS plus 1% Nonidet P-40 lysis buffer (PBSN), 2 μg flag antibody and IgG antibody were mixed in PBSN. Then, 50 μL 1% trifluoroacetic acid was added to wash the samples. After the supernatant was collected, samples were neutralized by the addition of 5 μL 10% ammonia, and proteins were eluted in 100 μL ammonium bicarbonate buffer. Next, 5 mM Tris (2-carboxyethyl) phosphine was used to reduce the samples, and 0.5 μg trypsin was added to obtain zymolized peptides. After desalination, peptides were detected using MS. From every group, a 5-μL peptide solution was separated using a nano-UPLC Easy-NLC1200 liquid phase system and detected using an online mass spectrometer (QExactive). MaxQuant software (version 1.5.6.0) was used for database searches and quantitative analysis of MS data. The protein database was UNIPROT_HUMAN/Chlorocebus sabaeus_2016_09, and the quantitative method was MS1 quantification. The quantitative results were statistically analyzed to identify corresponding enriched proteins (53).

### Statistical analysis.

All results are represented as the means ± the standard error of the mean (SEM). Where appropriate, comparisons were analyzed using Student’s *t* test with a *P* value of <0.05 being considered statistically significant. Measured values and the corresponding statistical significance are shown in the figure legends. Statistical results were analyzed using GraphPad Prism 6.0 software.

### Ethics approval and consent to participate.

All animal protocols were performed according to the guidelines and approved by the Institutional Animal Care and Use Committee of Academia Sinica.
