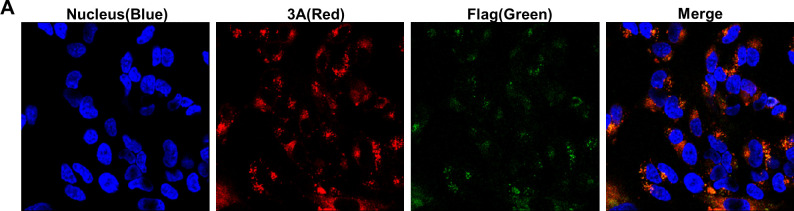# Correction for Wu et al., “Enterovirus A71 Promotes Exosome Secretion by the Nonstructural Protein 3A Interacting with Rab27a”

**DOI:** 10.1128/spectrum.01766-24

**Published:** 2024-10-18

**Authors:** Jing Wu, Yuxue Zhao, Qiaoqiao Chen, Yiwen Chen, Jiaqi Gu, Lingxiang Mao

## AUTHOR CORRECTION

Volume 11, no. 2, e03446-22, 2023, https://doi.org/10.1128/spectrum.03446-22. While preparing the data for Fig. 5A and S3B, we inadvertently swapped one fluorescence image with another due to their similarity. The published values are correct, and the conclusions of the paper remain intact. We apologize for this error.

Page 8: Figure 5A should appear as shown below.

Supplemental material: Figure S3B should appear as shown in the revised supplemental file in this author correction.

**Fig 5:**