# Supplementary material for: Enterovirus A71 Promotes Exosome Secretion by the Nonstructural Protein 3A Interacting with Rab27a
Source: Microbiol Spectr. 2023 Feb 15;11(2):e03446-22. doi: 10.1128/spectrum.03446-22 (PMC10101103; doi:10.1128/spectrum.03446-22)
Supplement: Supplemental file 1 — Supplemental material. Download spectrum.03446-22-s0001.pdf, PDF file, 0.7 MB [file spectrum.03446-22-s0001.pdf]

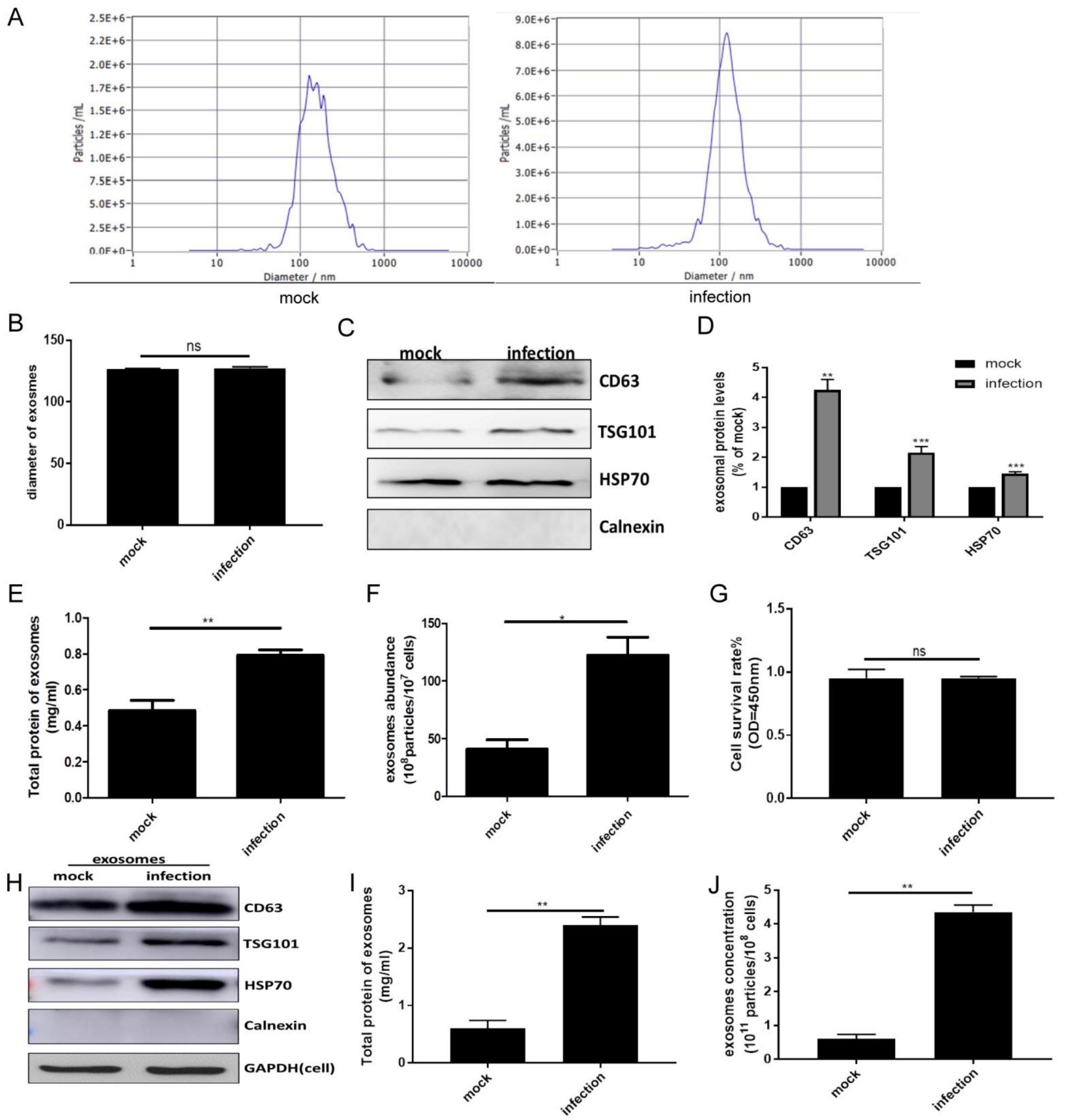

Fig. S1 EV-A71 infection promoted exosomes secretion in Hela cells. Exosomes were isolated and purified by serial centrifugation from supernatants ofEV-A71- and mock-infected HeLa cells (MOI: 1, post-infection time: 24 hours). (A) NTA analysis comparing exosomes particle numbers and size. (B) Statistical analysis ofexosomal diameter. (C) Exosomal marker proteins detected by WB analysis. (D) Quantitation of exosomal protein levels by Image J software. (E) Exosomal total protein content detected by BCA assay. (F) Abundance of exosomes detected using the ExoELISA kit. (G) Cell viability measured using a CCK8 kit. (H) WB analysis of exosome marker

proteins, (G) Total exosomalprotein contentand (H) The abundance of exosomes from EV-A71 -or mock-infected Hela cells (MOI：0.1, post-infection time：18 hours). (*p<0.05; **p<0.01; ***p<0.001). Data shown represents three independent experiments.

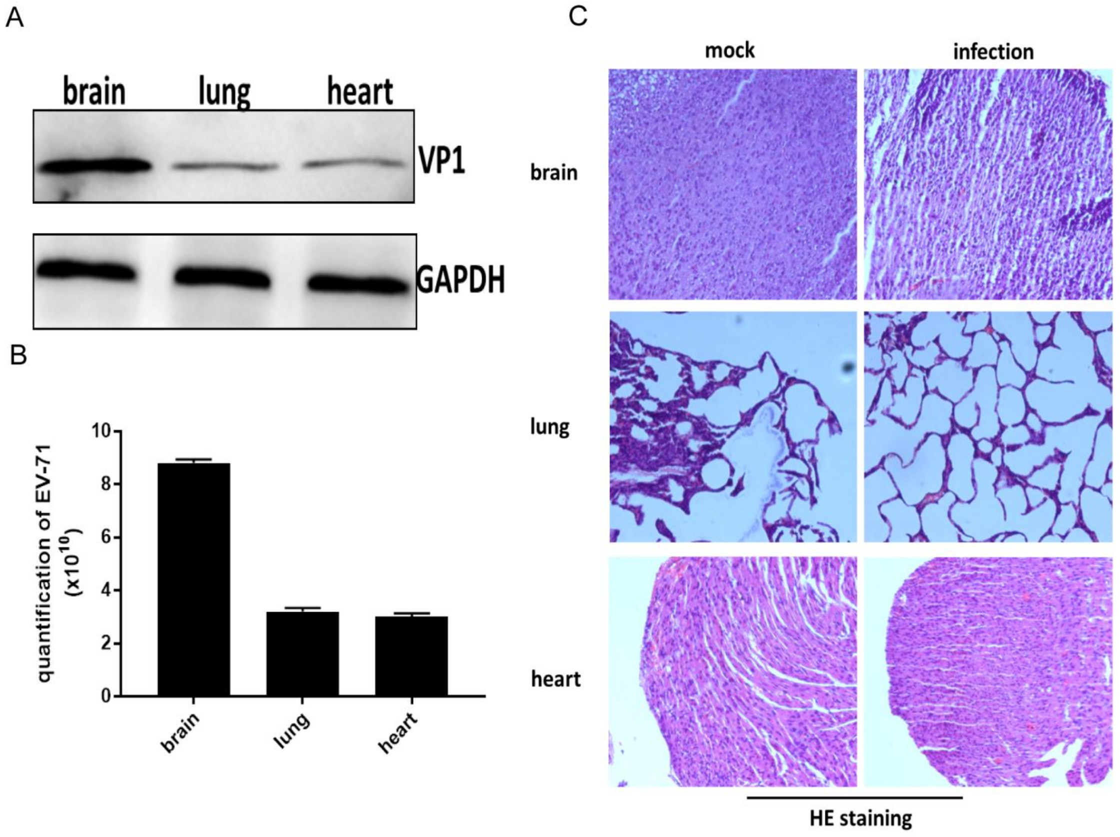

Fig. S2 EV-A71 proliferated at a different rate in various tissues. (A,B) Relative expression of the viral VP1 proteinand RNA in brain, lung, and heart tissues of EV-A71-infected mice detected by WB (A) and qRT-PCR (B)analysis, respectively. (C) Histology of brain, lung, and heart tissues, stained by hematoxylin and eosin (at ×10 magnification). Data presented represents three independent experiments.

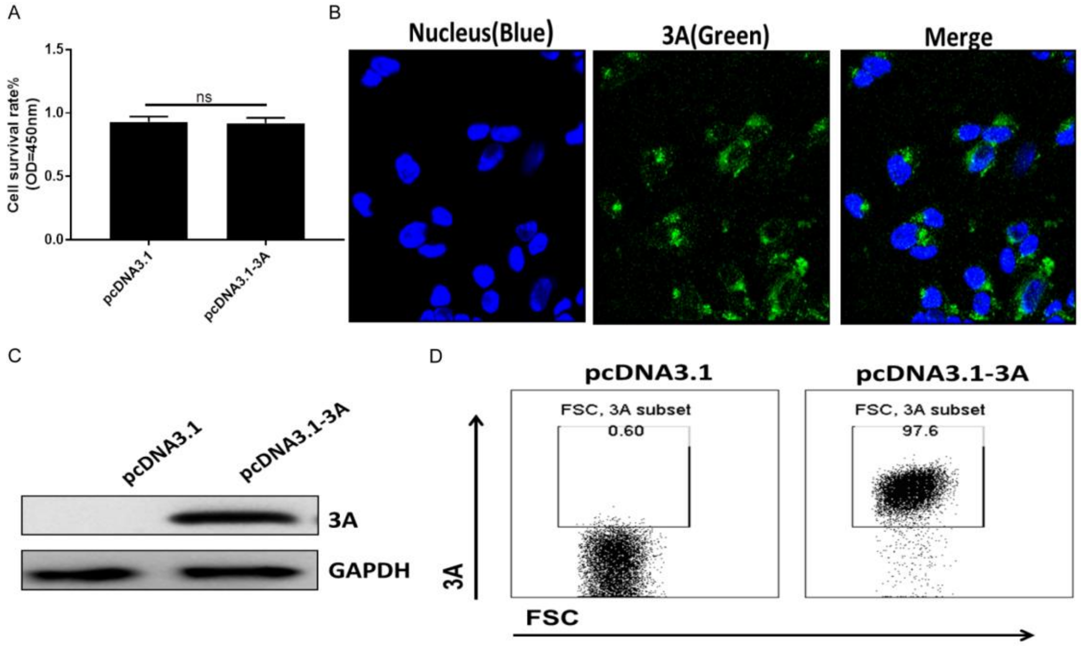

Fig. S3 Detection of stably expressed 3A protein in cells by WB，IF and flow cytometry. (A) Cells viability measured using a CCK8 kit.(B) Hela cells stably transfected with the vector encoding pcDNA3.1-3A. Cells were fixed and stained with an anti-3A (green) antibody. Fluorescence signal detected by confocal microscopy. Bar=20μm. (C) WB analysis of 3A with antibodies against 3A and GAPDH. (D) Detection of 3A expressing cells by flow cytometry analysis after staining with a FITC labelled anti-3A antibody. Data were obtained from three independent experiments. (ns= no significance)

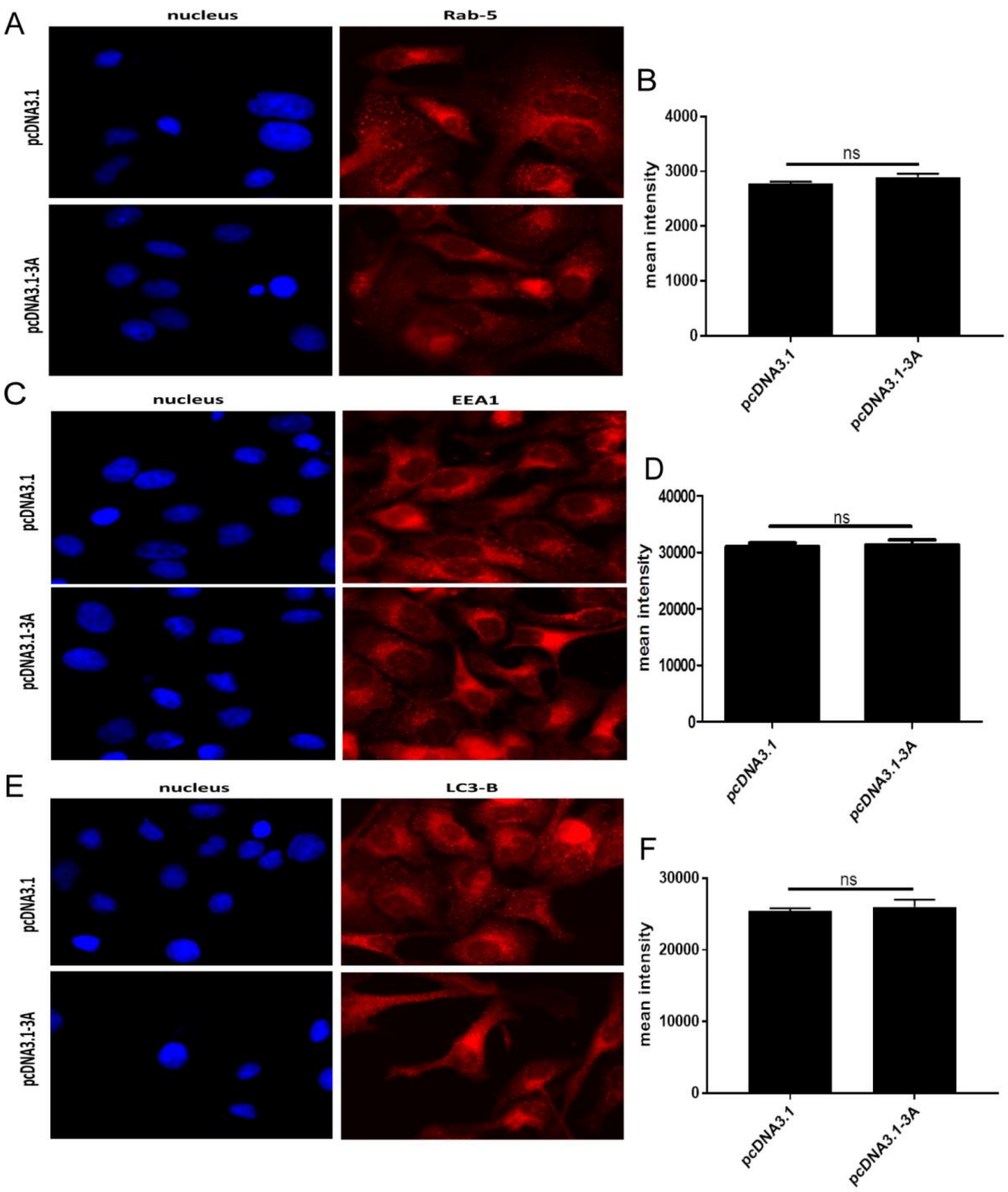

Fig. S4 Exogenous 3A have no significant effect on early endosomes or autophagosomes. (A, C) Representative images of two cells stained with antibodies against the early endosomal markers Rab5 and EEA1, imaged by confocal microscopy. (E) Confocal microscopy images of cells stained with an antibody detecting the autophasome marker LC3B. Red signals, Rab5, EEA1 and LC3B, blue 4′, 6-diamidino-2-phenylindole (DAPI) signal staining the nucleus. Scale bar = 10 μm. (B, D and F) Normalized expression of Rab5, EEA1, and LC3B calculated using the

endogenous protein GAPDH as a housekeeping protein. Analysis was carried out using the Image J software. Data presented from three independent experiments. (ns = no significance)
